# A New Departure

**Published:** 1900-03

**Authors:** 


### EDITORIALS.

### A NEW DEPARTURE.

THE modest invitation sent forth from the University of Pennsylvania Veterinary Department to the veterinarians in active practice in the State to come and spend a period of ten days in post-graduate instruction free of any expense has proven a new departure of great importance to the profession.

The success attending this initial effort far exceeded the most sanguine expectations of any one of those who proffered their services in conducting the work.

More than fifty active practitioners responded to the call, and with one accord voted that it be made an annual feature.

Clinical instruction daily in the hospital, lectures on every aspect of sanitary control work, laboratory demonstrations, visits to ideal dairy farms, talks on aspects of State and municipal laws and ordinances, and a thorough understanding of how these may be maintained, extended, and enhanced in their value to our people, sent forth at its close a body of men made better in every way for their work, each one a new centre of force to awaken his people to the value and need of this work and better enabled to perform the duties required by the State Live-stock Sanitary Board. A dinner given by the University at the Faculty Club's home added much to the fraternal feeling on this occasion, and the fruits of this undertaking will grow with each added year to the honor of Pennsylvania's earnest, aggressive, and progressive veterinarians.

Much credit, indeed, is due to State Veterinarian Pearson, who planned the project, and to Professors Adams, Harger, Ravenel, Hoskins, Conrad, and Klein, who gave their services in the work.

